# Oral health plays second fiddle in palliative care: an interview study with registered nurses in home healthcare

**DOI:** 10.1186/s12904-021-00859-3

**Published:** 2021-11-10

**Authors:** Anna Gustafsson, Johanna Skogsberg, Åsa Rejnö

**Affiliations:** 1 Home Health Care, Härryda municipality, Härryda, Sweden; 2 MedPro Clinic Stavre Primary Health Care Centre, Trollhättan, Sweden; 3 Department of Health Sciences, University West, Trollhättan, Sweden; 4 Skaraborg institute of Research and Development, Skövde, Sweden; 5 Department of Medicine, Skaraborg Hospital Skövde, Skövde, Sweden

**Keywords:** Community care, Content analysis, End of life, Home healthcare, Interviews, Oral health, Palliative care, Registered nurses

## Abstract

**Background:**

Oral health is crucial to the experience of well-being, and symptoms from the mouth are common at the end of life. Palliative care aims to identify and treat symptoms early to avoid unnecessary suffering and is thus an important part of nursing in home healthcare. The aim of this study was to illustrate the professional reflections of registered nurses about oral health amongst patients in palliative care, who are being cared for in a home healthcare setting.

**Results:**

The results showed oral health in end-of-life care, to be an area marked by responsibility and ethical considerations. This was seen in all four partly overlapping themes that emerged through the analysis: *Oral health is easily overlooked in palliative care, Oral health is everybody’s but in reality nobody’s responsibility, Patient integrity can be an obstacle for oral health,* and *Focus on oral health is urgently needed*. The mouth is often not included as part of the daily basic care routine, by the registered nurses and the home healthcare staff, until the patient is near end of life. Moreover, neither does the patient tell about symptoms from the mouth. The interpreted whole indicates that the registered nurses had a bad conscience about not doing what they are actually responsible for and ought to do.

**Conclusion:**

The oral health of patients at the end of life risks being forgotten or falling between the cracks, due to the nurses’ scattered tasks and unclear delimitations between their, and other professionals’ responsibilities. The responsibilities of registered nurses are also ethically demanding, since their intent to respect the patient’s integrity could mean that in some cases the patients does not allow them to help with oral health. To reduce the risk that oral health is overlooked, clearer demarcation and guidelines on the division of responsibilities are required. Routines that clearly implement early and recurring oral health assessments in home healthcare as well as continuing education updates on oral health and oral care are also needed.

**Supplementary Information:**

The online version contains supplementary material available at 10.1186/s12904-021-00859-3.

## Background

Oral health, an important component of general health, is crucial to our well-being whether healthy or ill. The mouth as a symbol has been described as “*the centre of many of the fundamental components of human activity*” and can be seen as *“a door or gate”* to a person’s soul “*which lends access to another realm of existence”* [1]*.* This emphasizes the importance of the mouth and of oral health in patients’ well-being in general, and particularly at the end of life. According to the definition by the FDI World Dental Federation, oral health is “*multi-faceted and includes the ability to speak, smile, smell, taste, touch, chew, swallow and convey a range of emotions through facial expressions with confidence and without pain, discomfort and disease of the craniofacial complex*” [2].

Palliative care is an area of increasingly global concern as a result of, among other things, an ageing population, and an increase in the incidence of chronic diseases [3]. More than 58.6 million people are estimated to be in need of palliative care every year, and about half of these (25.7 million) are near the end of life [4]. According to the WHO, [5] palliative care means an approach “*that improves the quality of life of patients (adults and children) and their families who are facing problems associated with life-threatening illness”.* Palliative care is part of the third UN sustainable development goal and needs to be improved not least at the primary care level in order to decrease avoidable suffering [3]. Palliative care aims to identify, assess and treat pain, and other physical, psychological, existential, and spiritual problems as early as possible. Sixteen symptoms, one of which is dry mouth, have been denoted to identify palliative care needs [4]. Oral health problems may affect all the above mentioned dimensions negatively, [6, 7] in everyday life and hereby affect the quality of life for patients with palliative care. Difficulties in talking, eating and being close to loved ones occur [7, 8] and it is therefore crucial that these problems are addressed.

Problems with the oral cavity that can arise at the end of life, in addition to dry mouth, include pain, changes in taste and changes in the mucosal membranes [8, 9]. As many as 78% of patients with cancer in the palliative phase experienced dry mouth as the second most troublesome symptom, after fatigue, as measured with the Edmonton Symptom Assessment Scale (0–10 p): 4.7 points compared to 4.9 [9]. Oral candidiasis is almost twice as common in patients at the end of life as in healthy individuals [10]. Dry mouth, inflammation of the tongue, dysphagia and spotting in the oral cavity have been reported to be more prevalent in patients nearing the end of life and symptoms like these can be used as indicators of a need for assistance with oral care from healthcare staff [11]. It has long been known that pathogens in the oral cavity contribute to pneumonia and that good oral care can reduce and prevent this [12]. Standard procedures for oral care can improve conditions in the oral cavity, symptom control and comfort amongst patients with advanced disease who need assistance to uphold oral health [13].

Despite the importance of oral health-related problems at the end of life, patients report that their problems are not always taken seriously by healthcare staff [7]. Patients have stated that healthcare staff seldom inspect the oral cavity and do not offer any help or relief of symptoms. This can lead to patients giving up reporting since there seems to be no ease of the symptoms. At the same time, patients also appear to diminish their problems with oral health through only “mentioning” instead of “emphasising” symptoms from the mouth [7]. Older patients in general appear to hesitate asking for assistance with oral care [14]. This combined with the lack of routine assessments of the mouth or healthcare staff’s incorrect performance of these assessments [15] contributes to the problem for healthcare staff to notice the need [16].

Difficulties in providing adequate oral care faced by healthcare staff, related to patient’s unwillingness to cooperate, exists in both hospitals and institutional facilities [16, 17]. Healthcare staff seem to be aware of the major impact oral health problems have on the experience of health at the end of life but lack of empathy or knowledge and low priority of these issues are seen as causes of the inappropriate care [16, 18]. To prevent oral health-related problems at the end of life, oral assessments need to be done regularly [8, 11]. These assessments can also contribute to detect the risk of aspiration pneumonia [19]. Using a comprehensive guide to assess oral health is beneficial as it provides a systematic assessment. A recent review found that many oral health assessment tools have poor scientific validity and reliability; however, the *Revised Oral Assessment Guide* (ROAG) and the *Oral Health Assessment Tool* (OHAT) were recommended as the best choice for assessment of the oral health of older people by non-dental professionals [20].

Being functionally dependent and thus needing assistance with oral care increases the risk of having poorer oral health [21]. Untreated oral problems were threefold higher in residents of nursing homes than in the community [21] and oral health in the elderly deteriorates after hospitalization [17]. This implies that carers, independent of the care context, appear to have difficulties in upholding oral health of the patients they are supposed to care for. Nevertheless, a Swedish study of people with dementia at the end of life indicated that oral health assessments were equally common in hospitals and nursing homes [22].

In Sweden, 36% of the population over 80 years receive home care in a community setting [23]. It has been concluded that most people want to die at home; however, they have been asked this question in advance and not at the end of life [24]. The proportion of people in Sweden who die in settings outside of hospital has increased; this proportion was 57.9% in 2012, with the likelihood of dying outside of hospital being higher for those with conditions “indicative of palliative care needs” [25]. In line with this, people with cancer are more likely to die at home than people with non-cancer diseases and, in most countries, the probability of dying at home also increases with older age [26]. This means that many frail patients with complex care needs at the end of life are cared for in a community care setting and that the management of several different care organizations need to cooperate regarding care of the patient. Cooperation in multi-professional teams, including registered and enrolled nurses, physicians and other professionals, is essential for promoting patients’ well-being and to facilitate dying [27]. Registered nurses (RNs) are primarily responsible for the nursing care and have an important role in leading and assigning care work and providing continuous tuition within the area of care at the workplace [28]. Information on the RNs perspective of oral health in patients receiving palliative care is scarce for settings outside of hospitals. Existing research indicates that oral health is neglected in patients receiving palliative care. This study intends to contribute to the discussion of oral health in patients receiving palliative care by adding reflections from RNs working in home healthcare on this issue.

### Aims

The aim of this study was to illustrate the professional reflections of RNs about oral health amongst patients in palliative care who are being cared for in a home healthcare setting.

## Methods

This qualitative study had a descriptive design [29], and used individual interviews to gain a deeper understanding of the professional reflections of RNs in home healthcare about oral health in patients receiving palliative care, in line with recommendations from the literature [30]. The Standards for Reporting Qualitative Research (SRQR) checklist was used (supplemental file 1).

### Setting

The interviews were conducted with RNs, with or without specialisation, who were working in home healthcare in a community setting in two municipalities in western Sweden. The RNs were working in ordinary housing, nursing homes or housing with special services for people with functional disabilities, hereafter referred to as “home healthcare”. The RNs were responsible for the care given to the patients, and led one or more groups of staff consisting of both enrolled nurses and untrained staff who were responsible for a certain area or nursing home. The expression “healthcare staff” will hereafter be used for the staff led by the RNs, regardless of their education. The RNs did not always take an active part in daily patient care and often acted as consultants when the healthcare staff needed advice about the care.

### Participants

In order to answer the research question purposive sampling was used [31]. Head managers of two municipalities in western Sweden gave their permission to interview RNs who were employed in home healthcare services, during their working hours. Contact information to RNs were obtained through their head manager and a letter of invitation to participate in the study was sent. The inclusion criteria were that they should be working as an RN in home healthcare and caring for patients at the end of life. RNs interested in participating contacted AG or JS to schedule an interview. Nine RNs from the two municipalities showed immediate interest in the study and were invited to participate. They were of mixed sex and age and had varied length of work experience (Table 1). Five were specialised as RNs and two were undergoing specialist education. The RNs had previous experience in various other care contexts, such as pre-hospital care, infection healthcare and palliative care. The interviews were performed during March 2019 in places chosen by the RNs themselves.

**Table 1:** Characteristics of participating registered nurses. Age, sex, number of years worked as RN and number of years worked in home healthcare

Age	Sex F/M	Years worked as RN	Years worked in home healthcare
44	F	26	9
53	F	24	13
50	F	25	21
45	F	22	5
55	F	33	10
33	F	1	1
59	M	32	3
56	F	32	27
45	M	16	10

### Data collection

The interviews were conducted by AG or JS. All interviews but one were carried out at the participants’ workplaces during working hours. The other interview was carried out in a secluded public meeting place during non-working hours. A semi-structured interview guide was used in order to gain a deeper understanding of the participants’ thoughts on oral health. The interview guide was constructed based on the literature, expert opinion and the authors’ clinical experiences [30]. The opening question was – “If we start with palliative care and I say ‘oral health’, what are your thoughts about that?” After that, there were follow-up and clarifying questions such as – “When you say oral health problems – can you tell me more about that?” The interview guide was used to ensure that the subject was covered but without any given order, thus allowing the interviewer to be sensitive to the interviewee’s path without losing the direction of the interview. During the interviews, we strove to get descriptions of oral health that were as multi-faceted as possible, in line with the recommendations of Kvale and Brinkmann [30]. Data collection were ended after the nine RNs that first showed interest had been interviewed since the data were considered rich enough to answer the aim. The interviews lasted 25–52 min (median 38 min), were digitally recorded, and were transcribed by the interviewer. Combined, they constituted 142 pages of transcribed data.

### Analysis

The data was analysed using qualitative content analysis based on the description by Graneheim and Lundman [32], since this method is considered suitable for analysing the content of narrated data. The analysis began with all the researchers reading through the transcribed interviews, first as a whole and then separately, to get an overall understanding of the content. After that, AG and JS separately identified and marked up the meaning units in the first interview. The mark-ups were compared, and considerable agreement was found. For text marked up by only one of the researchers (AG or JS), discussions were undertaken until consensus was reached. After that, each interviewer identified the meaning units of the interviews they had performed. Thereafter, all meaning units were condensed and given a label, in the form of a code giving information about its content, by AG and JS. The codes, which were kept on a manifest level close to the original text, were sorted according to differences and similarities in thoughts about oral health. The preliminary themes that were identified were tested and modified until consensus was reached by the research group as a whole. The analysis finally resulted in four themes which described the RNs’ reflections about oral care. An example of the analytical process is given in Table 2.

**Table 2:** Example of the analytical process with the text, meaning units, condensation, codes and themes

Text	Meaning units	Condensation	Codes	Themes
P: Yes exactly and maybe rather that they had not been asked before that it … felt strange I: Yes P: … mm but that one in such a late stage was asked about oral health	that they had not been asked before and it felt strange that one in such a late stage was asked about oral health	Patients felt that it was strange that they were not asked about oral health until such a late stage	Not being asked about oral health	Oral health is easily overlooked in palliative care
P: We have guidelines for everything else but I have never seen any memo or anything concerning oral health. I: That is interesting P: well … I am sure that we do it in different ways	We have guidelines for everything else but I have never seen any memo or anything concerning oral health; I am sure that we do it in different ways	Having guidelines for everything but oral health and never seen memos or anything about it	Guidelines for oral health are lacking	Focus on oral health is urgently needed

*I* interviewer, *P* participant

### Ethical considerations

This study adhered to the guidelines for empirical studies in Sweden. Ethical permission was given by the managers of each of the two municipalities that participated. No formal approval from the Regional Ethics Review Board was required for this kind of non-interventional study involving healthcare professionals which does not involve risks or the processing of sensitive personal data [33] and hence no ethical review was possible. Nevertheless, the work followed the ethical principles for medical research in the Declaration of Helsinki [34]. The RNs were given both oral and written information and written informed consent was obtained. The voluntary nature of participation and the possibility of withdrawing without prejudice were explained. The identities of the RNs are protected by the confidential handling of data in accordance with the EU regulation [35]. The data is stored on password-protected computers. All data will be archived for at least 10 years, according to the Archives Act [36].

## Results

Oral health at the end of life, from an RN’s perspective, was shown to be an area marked by responsibility and ethical considerations. This was seen in all four partly overlapping themes: *Oral health is easily overlooked in palliative care, Oral health is everybody’s but in reality nobody’s responsibility, Patient integrity can be an obstacle for oral health, and Focus on oral health is urgently needed*. The interpreted whole indicates that the RNs had a bad conscience about not doing what they are actually responsible for and should do.

### Oral health is easily overlooked in palliative care

The RNs described oral health in palliative care within home healthcare as a natural and important part of care to be performed regularly and frequently, but also as something that easily was forgotten. Structured oral health assessments and oral care might seem trivial, but the RNs considered they were more difficult to carry out than, for example, pain assessment. They were in agreement that oral health has a great impact on the patient’s overall health. Oral health was seen as an important part of holistic care, since it is always present and has a great impact on the patient’s well-being. The RNs also stated that impaired oral health affected the patients’ overall health and could spread like ripples, contributing to severe consequences in several other areas.

Despite the holistic view of their patients described by the RNs, it also emerged that the mouth was sometimes overlooked as a part of the body. The reasons for the patient’s symptoms were first sought outside the mouth. The patient’s oral health was also neglected because the subject was not always brought up in or included as a component of care. The oral health status were not always immediately apparent to an outside observer and could be ignored by patients, relatives and staff, leading to under-treatment of problems. The patients might also be unaware that ease of oral problems could be at hand and consequently did not ask for help.

“She is so very clear-headed, so one thinks ‘why don’t you ask for help with it’ (oral care)*” (IP7).*

The RNs also described how patients who were asked about their oral health reacted to the fact that they had not been asked earlier. When the patient’s health deteriorated and they became bedridden, stopped eating and drinking and breathed through their open mouth, the mouth became more visible and was thus more naturally seen as a part of the holistic care.

“It is actually easiest when the patient is in the final stage because then you have the opportunity. You are naturally there all the time, moisturizing, fixing, anointing and making sure the mouth is not dry” (IP3).

The RNs said that some nursing colleagues as well as other healthcare staff were not completely comfortable performing oral health assessments, but thought that this became easier and more natural when the patient was in the final stage of life. The mouth was then less likely to be forgotten.

### Oral health is everybody’s but in reality nobody’s responsibility

Although the RNs considered oral health at the end of life, including practical oral care and documentation of systematic oral assessments, to be their responsibility, it was evident that they sometimes used the exclusion method to come to this conclusion.

“It is not the responsibility of dental care practitioners, nor of the enrolled nurses, so it is probably our responsibility.” (IP2)

Oral care was not seen as an entirely natural part of the RN’s responsibility and was thus easily down-prioritized as a result of the many areas of responsibility that were considered important.

The patient’s oral health was described as a team effort, shared by several professions, and continuity and collaboration were considered important factors for success. The RNs stated that contact with other care providers was usually undertaken by them, and it was also they who, after assessment, initiated any eventual treatment. Dental care practitioners, hospital dental care practitioners and the palliative care team were seen as resources, while other inpatient care facilities and primary care physicians were considered to have insufficient or only generalized knowledge about oral health. Moreover, there were occasionally disagreements about treatment strategies between RNs and physicians since the RNs sometimes considered themselves as having more expertise within oral health and its treatments.

"We have a doctor, but she is not very familiar with “the mouth” (oral health) (IP5)".

The RNs described their profession in the home healthcare setting as “*a wandering profession*” and stated that they were dependent on other healthcare staff informing them about changes in the oral status or deterioration of the patient. It was thought that the healthcare staff, although considered skilled and experienced, not always performed structured oral assessments. The RNs led the working group and distributed the responsibilities for practical care to be performed while supporting, tutoring and acknowledging the healthcare staff but wished for more time designated to that. The RNs considered it their responsibility to convey confidence, and to encourage the healthcare staff to realise the importance of oral health and oral assessments and that the mouth is an important part of the body and should be an integrated part of the daily basic care routine.

“It's great to see, now that the youngsters have been working here for a few months, that (now when) they come to me when someone is not feeling well or has not eaten, they have checked! (the mouth) in the way I showed them" (IP1)

The RNs saw the relatives as part of the team around the patient and thought oral care could be used as a way to encourage the relatives to approach the severely ill person, especially if they were finding this difficult.

"Relatives may feel that they can’t help the loved one with anything, but ... if you give them small tasks (regarding oral care) it seems to become very positive.” (IP5)

The RNs perceived that relatives often found it comforting to be able to help but that they could also experience it as difficult and that the RNs needed to instruct and support them. It was thus very important to emphasize that the RNs still had the final responsibility for the patients’ oral health. If the relatives did not carry out any oral care, the patient would still get help with this from the healthcare staff and the RNs.

### Patient integrity can be an obstacle for oral health

Respect for the patient’s integrity could, according to the RNs, be an obstacle to oral health. In order not to violate the patient’s integrity, the work with oral health needs to be individualized. The individual adaptation of care, using a person-centred approach, was described as having to consider differences between individuals, for example age differences, differences in individual views on what is private, and differences in personality such as the loner. Sometimes patients did not want to receive assistance with oral care or assessment of oral health, despite the RNs’ consideration that this risked causing oral health problems. A delicate ethical balance was described between respecting the patient’s unwillingness to accept assistance with their oral health and the RN’s wish to do good for the patient.

“You believe you do something good when you let someone be, but that is not always the case (IP4)”.

The RNs also sometimes experienced internal ethical conflict when attending to a patient’s oral health in situations where the patient and the RN had no previous relationship and the patient could not give consent to the care as a result of unconsciousness or cognitive impairment.

"She was unconscious and could not say yes or no to it (oral care) so I felt that when you get into the mouth and check with the lamp and your fingers and so on, it felt a little uncomfortable" (IP6).

Emotional perceptions around the mouth were twofold; some RNs reported experiencing the mouth as a private and sensitive zone while others did not look upon the mouth as different from the rest of the body. It was thought that the healthcare staff could have the same dual perceptions about the mouth.

"Some home healthcare staff consider the mouth so intimate, they have no problem wiping someone's behind but have a very hard time looking into someone’s mouth" (IP7).

The RNs said that also patients might feel that the mouth is an intimate area and related to integrity, leading to an unwillingness to open their mouth. Patients’ feelings of fear and panic or of experiencing oral care as uncomfortable and difficult to endure were also mentioned. It was considered to be of the utmost importance that patients did not experience oral care as abusive. The RNs described situations where patients experienced it as painful and time-consuming to have their oral health assessed and any complaints remedied. They put this in relation to the risk of ill health if the problems were not detected and relieved.

The RNs said that they and the healthcare staff helped to find strategies for preserving the integrity of patients who did not want to open their mouths. The strategies, such as reasoning with the patient about being allowed to carry out oral care or trying to look into the mouth from a distance, differed according to whether or not the patient was competent to make decisions. Not being allowed to help with the patient’s oral health problems was described as frustrating and the RNs had to accept that they could only do what was possible.

“Then we must realise that we cannot succeed every time with this group of patients. But you may succeed five times out of ten, which is much better than nothing” (IP1).

The RNs also described other ethically demanding situations where, for example, relatives could not understand why the staff did not force the patient to succumb to oral care, as may have occurred earlier when the relatives were helping the patient. The RNs described that part of their role was to explain the situation to the patient and relatives, and also to the healthcare staff.

### Focus on oral health is urgently needed

The RNs described a great need to focus on oral health in order to uphold the quality of the offered care. Oral health was perceived as being easily forgotten if it was not constantly paid attention to. The RNs stated that, by increasing the focus on oral health, they had become better at paying attention to it in their duties, but thought that there was still room for further improvement. Data from quality registers could contribute to increased attention paid to oral health, could highlight areas for improvement, and could allow for results to be followed over time.

“The palliative care register accounts for certain things. You want to reach up to set goals ... now the focus is on oral health assessments and so it also becomes more important to make the assessments and to document them” (IP2).

However, it was emphasized that quality registers were of little use if oral care was not carried out as decided for each patient.

The RNs emphasised the importance of commitment from the management and of communication between the RNs and the management. The management could accordingly both support and hinder focus on oral health. It was also thought that colleagues in coordinating key positions, such as palliative coordinators, could provide support and contribute to increased focus on oral health, while the lack of uniform routines and guidelines was mentioned as obstacles.

“We have guidelines for everything else but I have never seen any memo or anything concerning oral health” (IP7).

Knowledge of the individual patient’s oral health was stated as a key aspect of the work to prevent oral health problems but since the level of knowledge about oral health among the healthcare staff was considered to have decreased in recent years it could be difficult to get this information. Training in oral care and having access to the appropriate equipment could help to encourage oral care and make it more visible. The current undergraduate education of RNs was generally described as providing only limited, and mostly practical, knowledge of oral health.

“I have to think … Did we learn about the mouth (during nursing education)? … We ‘passed by the mouth’, I can say. But… the mouth itself – no” (IP6).

Some higher education institutions were stated to currently include oral health in the education programmes for nursing students, as seen in the approach of newly graduated RNs to the mouth as part of the patients’ daily bodily care. It was stated that integration of oral care into education would raise both awareness of oral care and the status of performing it. Continuing education was given in the workplace but the RNs wished for it to be more extensive than currently offered.

"Before my education (as a registered nurse), I thought someone else could do it (oral care), it was not a prioritized task for me” (IP8).

The RNs stated that when systematic assessments were performed (e.g. with ROAG), this led to both a focus on oral health and to uniform assessment of all parts of the mouth. This in turn made it easier to detect oral health problems while also providing suggestions for further actions. Difficulties implementing ROAG in the home healthcare group compared with implementing assessment instruments regarding, for example those used for nutrition and pressure ulcers, were described by the RNs. However, if an instrument was used regularly, it was considered that the staff would feel more confident in using it and thus benefit even more from its utilization.

## Discussion

This study reports reflections of RNs on the oral health of patients at the end of life receiving home healthcare within the community setting, where responsibility and ethical considerations characterize the results. Despite the RNs united view that oral health is an important part of the patient’s overall health near the end of life, the theme *“Oral health is easily overlooked in palliative care”* revealed that the perceived importance is not always visible through the RNs’ caring acts. The RNs’ reflections showed that oral health is easily forgotten, missed or lost among their scattered assignments, in line with previous research showing negligence with respect to oral health [37, 38]. The present study indicates that the neglect of oral health in these patients is mainly related to the multifaceted nature of the responsibilities of the nursing profession in this field, as opposed to other studies which have pointed out lack of time as a major cause [37–39]. Could the stated argument “lack of time”, as found in previous research, been used as an evasion, a method to avoid confronting the neglect of oral health? Perhaps lack of time is seen as a more legitimate reason for losing sight of oral health than admitting failure or even a conscious avoidance of the responsibility. There were indications in the present study that the RNs had a bad conscience about not doing what they are actually responsible for and obliged to do. It could be speculated that this bad conscience might be the reason behind arguments about lack of time in other studies. The present study clearly does not support lack of time as an argument, but instead indicates that there are more comprehensive causes related to personal responsibility. There were also indications in the present study, as found elsewhere [38], that our attitudes to our own mouth affect our view of oral health; however, practical experience of impaired oral health in patients appeared to be more important, implying a greater focus on responsibility regarding patients’ oral health.

A revised definition of palliative care has been proposed [40] to replace the WHO definition from 2002. The number of patients considered to be eligible for palliative care could potentially increase as a result of the revised definition stating that “*palliative care is the active holistic care of individuals across all ages with serious health-related suffering”* [6]. Since health-related suffering is considered serious when *“it cannot be relieved without medical intervention and when it compromises physical, social, spiritual, and/or emotional functioning*” [6], it becomes even more important to emphasize easing oral health problems in home healthcare.

The theme “*Oral health is easily overlooked in palliative care*” shows the importance of focusing on oral health early, preferably as part of the enrolment routine when initiating home healthcare, in order not to lose track of this important issue. It was also apparent that some patients might be unaware that oral health problems can be eased and therefore don’t ask for help. Previous palliative care research has found that patients might relate to the mouth differently from the way they relate to the remainder of the body; they will often only talk about symptoms from the mouth as an aside (“by the way...”) instead of reporting them specifically [7]. Awareness among RNs about the possible existence of oral health problems, even if the patient does not tell about symptoms from the mouth, could be an important piece of the puzzle. This clearly shows the value of not only an initial assessment of oral health, but also of regular follow-up assessments, allowing for early treatment or other measures if deficiencies in oral health are observed.

The RNs acknowledged the patient’s oral health as their responsibility, seen in the theme *“Oral health is everybody’s but in reality nobody’s responsibility”.* At the same time, they stated that the responsibility was not solely theirs and that the borders between and demarcation of their responsibilities were considered unclear. The responsibility of RNs for daily basic bodily care of patients is shared with healthcare staff such as enrolled nurses and care assistants, while the medical responsibility is shared with the physicians. The responsibility is also shared with dental health professionals, primarily responsible for the teeth but also partly responsible for the health of the rest of the oral cavity. According to the findings, oral health is everybody’s responsibility and the whole team’s effort is therefore crucial. Similar findings have previously been reported from within specialised palliative care, where the only professionals seen to fully acknowledge responsibility for oral care were the hospital dentists [16]. However, for dentists to be able to take that responsibility, they need to be alerted to the existence of patients’ oral health issues. Even if dentists are ultimately responsible for oral health, it cannot be their responsibility to perform practical daily oral care routines for the patients, not even at the end of life. This responsibility needs to be taken by those performing all other basic bodily care routines for the patients, such as RNs, enrolled nurses and care assistants.

As long as a person is healthy, the mouth is generally considered their own responsibility and the person takes care of their oral care themselves, including keeping contact with dental care providers. Even Swedish laws concerning health separate the mouth from the rest of the body by providing separate laws for the two areas: the Health and Medical Services Act [41] and the Dental Care Act [42]. When patients lose the ability to take care of their own oral health, there can be a sense of shame, sometimes from their own judgment but sometimes also from others, which could prevent them from asking for help. When a person needs support for their personal care and becomes a “patient”, there seems to be an (unconscious?) expectation that they will continue to take care of their own mouths in the same way as they did earlier in life. In palliative care, the care needs change seamlessly with time, and the need for oral care is often not as immediately evident as, for example, the need for help with intimate hygiene. The uncertainty surrounding oral health in palliative care means that patients, relatives or healthcare staff are required to draw the RNs’ attention to the fact that the patient has problems with their oral health or that the problems from the mouth become overtly noticeable before countermeasures are taken [16]. As shown in the theme “*Oral health is easily overlooked in palliative care”*, it might not be until the patient is bedridden, or even unconscious, with their mouth wide open, that the RNs’ expectation that the patient is fully responsible for their own oral health seems to cease. This is the moment when their full responsibility seems to dawn on the RNs, leading to actions being taken. Although this has been confirmed by others [38], research has also indicated there is a risk that oral care continues to be disregarded, even near the end of life [37].

The theme “*Patient integrity can be an obstacle for oral health”,* suggests that a patient’s unwillingness or inability to cooperate might be an obstacle to the RNs carrying out oral care and thus impeding the upholding of oral health, is in line with previous findings [16]. Failure to respect the patient’s integrity violates the patient’s autonomy and may even risk violating their dignity of identity [43]. One of the participating RNs stated that “My mouth is mine” emphasizing the intimate status of the mouth. This could help explaining the RNs experience that the patients’ integrity was more easily violated in connection with oral care than with other nursing care. Experiencing the mouth as potentially different from other parts of the body from an integrity perspective is clear from the results. The fact that patients can pinch their mouths and thereby show disinclination could be interpreted as an expression that they protect their personal integrity and could help explain the RNs experience that it is more difficult to perform oral care, than to perform intimate hygiene, against a patient’s will. It is important that care is not experienced as a form of abuse by the patient, even if the result would be objectively beneficial, and with a good relationship between the staff and the patient. These properties were considered key factors in promoting oral health for the patient in line with results by Ek & Browall [38]. To notice and be aware of a nursing care problem that could be eased by care actions, but not to be able to provide that care, can be truly challenging for RNs from an ethical perspective. In the present study, this was sometimes even considered to constitute an ethical dilemma for the RNs, which could contribute to a bad conscience.

The results emphasise that “*Focus on oral health is urgently needed”*. One issue was that oral health has not been seen as a main question by either caregivers or educational institutions (basic and higher education). This is despite both national organisations, such as the National Board of Health and Welfare, and international organisations, such as the Worldwide Hospice and Palliative Care Alliance, highlighting this as an area of importance and considering it a priority. The RNs also raised that it is important to have proper knowledge concerning oral health for the subject to be addressed. The lack of both awareness of, and education about, oral health within palliative care has previously been targeted in research [16, 37, 38, 44], and described as resulting in health professionals turning “a blind eye on oral care issues” [16]. In an intervention for RNs in the form of a two-day education on end-of-life the participants ranked knowledge about oral health as one of the most important gains [44]. Here, key areas for improvement are clearly seen.

For oral care in palliative settings and end-of-life care to take place and attract more attention, it seems reasonable to suggest an increased focus on oral health within healthcare education curricula, at both basic and higher levels, and on a continuing basis in the work place, in line with previous suggestions [38]. The RNs in the present study also called for memos and guidelines concerning oral health in palliative care since they are conspicuous by their absence, as noted earlier [16, 37]. If these measures were undertaken, healthcare staff would possibly have more knowledge, increased awareness as well as tools to handle oral health problems. All these put together would have the potential to increase the probability that oral health is given better and more systematic attention at the end of life within home healthcare.

### Strengths and limitations

This study addresses professional reflections on oral health, an area commonly causing unwanted symptoms in patients’ end of life, from RNs working in community home healthcare. The interview data were of good depth and provided a wide variety of descriptions of oral health at the end of life. The open interview format gave the RNs the possibility of developing their thoughts about oral health, thus contributing to the strength of the study. They willingly shared their thoughts and were open with their own faults and failings indicating a permissive climate during the interviews.

The diversity among the participating RNs is a strength of the study. Both men and women were represented, and there were different ethnicities and varied length of professional experience. The impact of experience and working time on the nurses’ views on and expertise within oral health is unclear. Longer experience of work as an RN will most likely give opportunities to encounter patients with oral health problems. The combined experience in healthcare for the group was well over 200 years (211 years), and almost half (99 years) of this was in the context of home healthcare, which can be considered a strength. However, personal interest in the field might also be important for developed reflections and expertise. While the sex distribution was uneven, it accurately reflects the actual gender distribution among professionals in healthcare. However, only RNs from a defined and similar socioeconomic area in Sweden participated, which is a limitation.

Methodological trustworthiness was achieved by carefully following the steps for content analysis as described in the methods section [45]. The first and second authors (AG and JS) performed the initial analysis, and the findings were then discussed in the research group until consensus on thematization was achieved. Trustworthiness within the study was further ensured by the three researchers discussing the analysis of the material grounded in our common understanding from home health care and our differing pre-understandings from acute hospital care (AG), primary care (JS) and acute stroke care and teaching of palliative care to undergraduate nursing students and graduated nurses (ÅR). However, the researchers were, generally speaking, from similar social and cultural backgrounds, which limits the interpretative framework of the analysis. The aim of including extensive quotations was to give the readers the possibility to judge the trustworthiness and the thematization of the findings. RNs from both the participating municipalities claimed that their organisation had extra focus on oral health in daily work, but each municipality in a different way. This means that reasoning about oral health might be reflected on or focused yet less in other home healthcare settings. Despite the different ways to focus on oral health in the two municipalities, the RNs reported similar results and shortcomings in execution. Accordingly, we suggest that the results of this study can be transferred to palliative care in other contexts within community care, and also to other contexts outside of specialised palliative care units where patients are cared for at the end of their lives. The results could also be of relevance to both early and late stages of palliative care and thus be transferable to most contexts where patients with serious health-related suffering are cared for.

## Conclusions

Oral health risk being overlooked or falling between the cracks. Despite oral health affecting the patient’s physical, social and emotional functioning yet the involved seem to overlook it and the problems associated with it. The wide variety of tasks assigned to RNs and the lack of a clear demarcation between the responsibilities of RNs and other professionals in this area are some of the factors that contributes to the overlooking of oral health. Other influencing factors are situations when patients no longer can carry out their own self-care, resulting in ambiguity regarding exactly when responsibility passes to other actors around the patient. We also show that ethical aspects influence the work with oral health. Respect for the patient’s integrity might lead to decisions that are in opposition to the ambition of the healthcare staff to do good, meaning that they feel they are not allowed to help the patients. This can result in a bad conscience and ethical stress for both RNs and healthcare staff.

It is important that oral health is attended to in order to fulfill the goal of palliative care: to relieve suffering and thereby improve or maintain quality of life in patients and their families who are facing problems associated with life-threatening illness. If oral health problems are overlooked, we lose the opportunity to relieve the suffering oral health problems might result in. To reduce this risk, clearer demarcation and guidelines on the division of responsibilities are required, as well as clear routines that implement early and recurring oral health assessments in home healthcare. With this increased focus on oral health, continuing education is also required in order for oral health not to be overlooked in home healthcare at the end of life. Further research on the views of the patients themselves on oral health at the end of life would be welcome.

## Supplementary Information

**Additional file 1.**

## Data Availability

The datasets analysed during this study are not publicly available because of the risk that individual privacy could be compromised; however, the datasets are available from the corresponding author on reasonable request.
